# Follow-up of an Intervention to Reduce Dental Caries in Indigenous Australian Children: A Secondary Analysis of a Randomized Clinical Trial

**DOI:** 10.1001/jamanetworkopen.2019.0648

**Published:** 2019-03-15

**Authors:** Lisa M. Jamieson, Lisa G. Smithers, Joanne Hedges, Jacqueline Aldis, Helen Mills, Kostas Kapellas, Herenia P. Lawrence, John R. Broughton, Xiangqun Ju

**Affiliations:** 1 Australian Research Centre for Population Oral Health (ARCPOH), Adelaide Dental School, The University of Adelaide, Adelaide, South Australia; 2 School of Public Health, The University of Adelaide, Adelaide, South Australia; 3 Dental Public Health, Faculty of Dentistry, University of Toronto, Toronto, Ontario, Canada; 4 Department of Oral Diagnostic and Surgical Sciences, Faculty of Dentistry, University of Otago, Dunedin, New Zealand; 5 The Ngāi Tahu Māori Health Research Unit, Department of Preventive and Social Medicine, Dunedin School of Medicine, University of Otago, Dunedin, New Zealand

## Abstract

**Importance:**

Testing the long-term usefulness of a childhood intervention and determining the best age of implementation are important for translation and policy change.

**Objectives:**

To investigate among children aged 3 years the long-term effectiveness an intervention that aimed to reduce dental caries among South Australian Aboriginal children and to assess if children in the delayed intervention (DI) group had any benefit from the intervention from ages 2 to 3 years and if the intervention usefulness was greater when delivered between pregnancy and age 2 years (immediate intervention [II] vs ages 2 to 3 years [DI]).

**Design, Setting, and Participants:**

Secondary analysis of a randomized clinical trial. The study enrolled 448 pregnant women across South Australia, Australia, at baseline (February 1, 2011, to May 30, 2012), with 223 randomly allocated to the II group and 225 to the DI group. Three-year follow-up data were collected November 2014 to February 2016.

**Interventions:**

The intervention comprised dental treatment to mothers, fluoride varnish application to children, and motivational interviewing delivered together with anticipatory guidance. This was delivered during pregnancy and at child ages 6, 12, and 18 months for the II group and at child ages 24, 30, and 36 months for the DI group.

**Main Outcomes and Measures:**

The mean number of decayed teeth measured at child age 3 years.

**Results:**

There were 324 children at age 3 years (52.3% male). The mean number of decayed teeth at age 3 years was 1.44 (95% CI, 1.38-1.50) for the II group and 1.86 (95% CI, 1.89-2.03) for the DI group (mean difference, −0.41; 95% CI, −0.52 to −0.10). The predicted mean number of decayed teeth at age 3 years for the DI group was 2.15. Between ages 2 and 3 years, the caries increment for the II group was 0.82 (95% CI, 0.75-0.89), compared with 0.97 (95% CI, 0.87-1.17) for the DI group (*P* = .05).

**Conclusions and Relevance:**

At the 3-year follow-up, II children had less dental caries than DI children, DI children developed dental caries at a lower trajectory than predicted had the intervention not been received at ages 2 to 3 years, and the caries increment was less between ages 2 to 3 years among II children compared with DI children. This study suggests that the best time to implement the intervention is earlier rather than later infancy.

**Trial Registration:**

Australian and New Zealand Clinical Trial Registry Ideintifier: ACTRN12611000111976

## Key Points

**Question:**

Does an early childhood caries intervention among Aboriginal Australian children have long-term effectiveness?

**Findings:**

At the 3-year follow-up of a randomized clinical trial involving 448 mothers and their children, children who received the intervention during pregnancy or early infancy had statistically less clinically detected untreated dental caries than their counterparts in the delayed intervention group.

**Meaning:**

A multipronged, culturally safe early childhood caries intervention delivered earlier rather than later in infancy may confer greater benefits among this population.

## Teaser

This secondary analysis of a randomized clinical trial investigates the long-term effectiveness of an intervention that aimed to reduce dental caries among South Australian Aboriginal children.

## Introduction

Poor oral health in childhood is socially patterned.^1^ It is a reflection of the social determinants of health and of the structure, access, and policies of dental health service providers. Dental disease in childhood may contribute to poor nutrition, alter ability to sleep and learn and play, negatively influence quality of life, and lead to increased financial stress in the family. Poor oral health in childhood is 1 of the key determinants of poor oral health in adulthood.^2^ A key global health target is reducing its prevalence.^3^ Most public health interventions to date that aim to reduce early childhood caries have focused on healthy diet,^4^ application of therapeutic agents that include topical fluorides and antimicrobials,^5,6^ effective toothbrushing,^7^ and behavioral strategies.^8,9^ However, these approaches may be insufficient because early childhood caries is more than just a biological response to sugar; it involves both biological and social constructs. To our knowledge, the strategies demonstrated as being effective have not been combined and tested in a programmatic approach. Also, to our knowledge, there has also been neither long-term follow-up of these interventions nor a test to demonstrate at what age in childhood the interventions might have the most effectiveness.

Indigenous Australians identify as being of Aboriginal and/or Torres Strait Islander descent. In the 2016 Census, they represented 3% of the total Australian population.^10^ Indigenous children aged 0 to 4 years represented 11% of the total Indigenous population, while similarly aged non-Indigenous children represented 6% of the total non-Indigenous population.^10^ Owing to sustained government policies of forced child removals, discrimination, and disempowerment, contemporary Indigenous Australians reflect 1 of the most disenfranchised groups at an international level. In 2015, Australia ranked second (after Norway) in the Human Development Index.^11^ However, this ranking dropped to 122nd when Indigenous Australian populations were separately analyzed.^11^

Indigenous Australian children score lower on most indicators of general health and well-being compared with non-Aboriginal Australian children.^11,12,13,14,15,16^ The literature suggests that many of the conditions experienced in Indigenous childhood are antecedents to chronic disease in later life.^17^ Dental disease in Indigenous Australian children is common and, in many cases, severe. It frequently causes acute distress, with care under a hospital-based general anesthetic being the only treatment option available. In the National Child Oral Health Survey, 44% of Indigenous children had 1 or more deciduous teeth with untreated dental caries compared with 26% of non-Indigenous children.^18^ Indigenous children in some locations have dental disease levels that are 5 times that of non-Aboriginal children.^19^ Lack of access to service providers is frequently cited as a reason for this inequity together with specific behavioral risk factors and social determinants.^20^

Results have previously been reported from an early childhood caries intervention (Baby Teeth Talk by the trial’s Aboriginal Reference Group^21^) among Indigenous Australian children, which comprised provision of dental treatment to mothers during pregnancy, application of fluoride varnish to teeth of children (at ages 6, 12, and 18 months), and motivational interviewing (MI) delivered together with anticipatory guidance. The primary outcome was reported at child age 2 years, at which time the mean number of decayed teeth (mean dt) was significantly less in the intervention group compared with the control group. After the 2-year follow-up, the control group received the intervention (a requirement of our ethics committee), with a further follow-up of both groups conducted at child age 3 years. This provided an opportunity to test (1) if the usefulness of the intervention among the immediate intervention (II) group persisted at the 3-year follow-up, (2) if the predicted trajectory of untreated dental decay in the delayed intervention (DI) group decreased after the intervention from ages 2 and 3 years, and (3) if the intervention was more effective in pregnancy or early infancy compared with later infancy or early childhood.

Our hypotheses were tripartite. These included (1) that consequences of the trial would be demonstrated at the 3-year follow-up (children in the II group would have fewer teeth with untreated dental caries than children in the DI group), (2) that children in the DI group would have some benefit from the intervention from ages 2 to 3 years (the predicted trajectory of teeth with untreated dental caries decreased), and (3) that the intervention would be more effective (ie, the increment in untreated dental caries between the 2-year and 3-year follow-ups) in pregnancy or early infancy (II group) than in later infancy or early childhood (DI group).

## Methods

### Background

**Figure 1.**
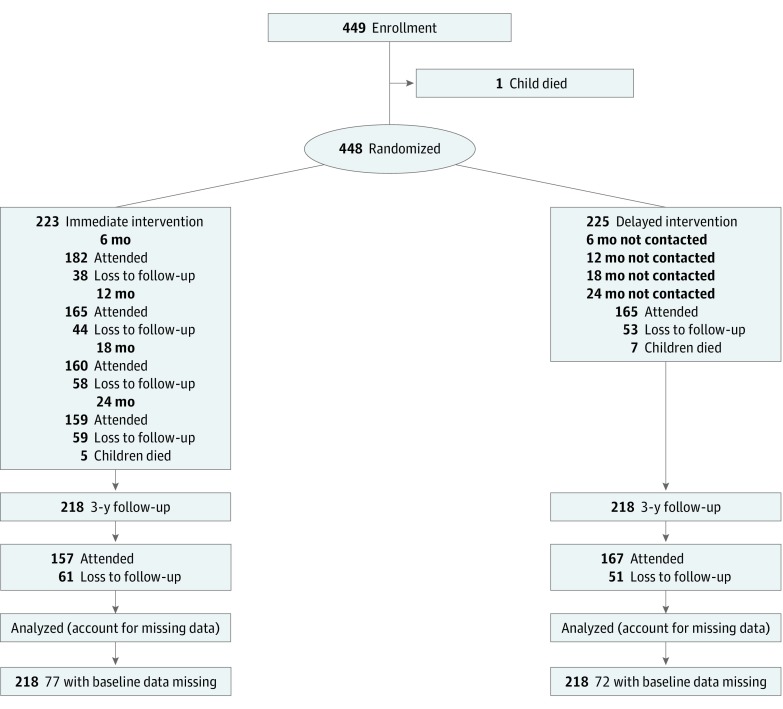
Flow Diagram of Participants Through Key Stages of the Randomized Clinical Trial

This study was a 1-year follow-up to Baby Teeth Talk, a 2-arm parallel, outcome assessor–masked, randomized clinical trial conducted in South Australia, Australia (Figure 1).^21^ The primary outcome of the original trial was the mean number of teeth with untreated dental decay in children at age 2 years. The trial protocol for the initial study was previously published.^22^

The ethical standards of the Declaration of Helsinki^23^ and Good Clinical Practice guidelines were followed for both the original trial and the follow-up. The University of Adelaide Human Research Ethics Committee, the Aboriginal Health Council of South Australia, the government of South Australia, and the human research ethics committees of participating South Australian hospitals all provided ethical approval for the study. This study followed the Consolidated Standards of Reporting Trials (CONSORT) guidelines.

### Participants and Follow-up

Baby Teeth Talk enrolled women who identified as being pregnant with an Aboriginal and/or Torres Strait Islander child between February 1, 2010, and May 30, 2011. Three-year follow-up data were collected November 2014 to February 2016. The subsequent sample comprised two-thirds of those who were eligible during the recruitment phase and was representative by maternal age, socioeconomic characteristics, and tobacco smoking status.^21^ Recruitment was mainly through the antenatal clinics of South Australian hospitals and through Aboriginal Community Controlled Health Organisations. All participants provided written informed consent. We randomly allocated 448 women to either an II group or a DI group.

The II group received 4 interventions. First was provision of dental care to mothers during pregnancy. Publicly funded dental care through South Australia’s Dental Service (SADS) was provided to mothers allocated to the intervention arm who held a government means-tested health care card. Study staff, in conjunction with the SADS’s Aboriginal Liaison Program, coordinated transportation and appointments. Six dental providers in the private sector, who were partners in the project, provided care to participants who were not eligible for publicly funded dental care (no costs incurred to the participant). Although cosmetic dentistry, endodontics, or orthodontics was not included, participants did receive, where necessary, extractions (including wisdom teeth), restorations, scale and cleans, radiographs, and checkups. Second, at child ages 6, 12, and 18 months, fluoride varnish was applied to teeth. The fluoride varnish protocol was adapted from that created by Slade and colleagues.^24^ Study staff who had been trained in its use applied the varnish. The child’s head would be on the lap of the study staff, with a knee-to-knee position adopted. Fluoride varnish was applied beginning with the back teeth (if present) and then progressing forward to the front teeth (after teeth had been cleaned and dried with gauze). It was suggested to caregivers to not provide the children with food or drink for the half hour immediately after fluoride varnish application. Third was anticipatory guidance. This occurred in conjunction with the MI during pregnancy and when children were aged 6, 12, and 18 months. The educational packages were tailored to include explicit oral health information relevant to pregnancy gingivitis and dental care provision (delivered during pregnancy), focus on first solid foods on eruption care for baby teeth (child age 6 months), focus on fluoride and toothbrushing and avoiding food and beverages with high sugar levels (child age 12 months), and focus on eruption of molar teeth at the child’s first dental checkup (child age 18 months). Fourth was MI. As mentioned, the MI component was delivered in conjunction with anticipatory guidance. A basic 2-day MI training course was attended by all study staff, followed by a 1-day follow-up course that was more intense in its delivery and purpose. One-day follow-up courses continued monthly for 6 months, followed by bimonthly single-day coaching and telephone coaching, when needed, for another year. The sessions were customized to meet the needs of each participant, with each session conducted on a one-to-one basis in participants’ homes or other venues where participants felt comfortable (eg, Aboriginal health services, libraries, and community halls). Each session lasted around 30 to 90 minutes. As recommended by Venner and colleagues,^25^ pictorial prompts and plain English were used. Fidelity was acceptable^25^ and was assessed by a member of the Motivational Interviewing Network of Trainers.

Mothers in the DI group received dental care when their child was aged 24 months. Fluoride varnish application to the teeth of the children, anticipatory guidance, and MI were delivered at child ages 24, 30, and 36 months, respectively.

Oral examinations were conducted by calibrated and masked examiners at child ages 24 and 36 months. These examiners followed a standardized protocol to record dental disease experience, which included predecayed and decayed (noncavitated plus cavitated lesions), missing, and filled surfaces. Children were examined in the knee-to-knee position on their mother’s lap, as is considered appropriate for this age group. Teeth were dried with cotton pads before examination. The light source was a fiber-optic light, and standard infection control procedures were followed. Only visual criteria were used to assess diagnoses, with measures including untreated dental caries, teeth missing owing to dental disease experience, and teeth filled owing to dental disease experience. The SADS was the provider referred to when any child was diagnosed as having untreated dental caries.

### Outcomes

In the present analysis, the primary outcome was the mean number of teeth with untreated dental caries in the child at a mean age of 3 years. Dental decay was computed at the threshold of precavitation, an area of demineralization without loss of surface continuity and cavitation (demineralization) (ie, a dental caries–caused break in the enamel surface). We also examined the mean levels of missing and filled teeth, as well as the prevalence of untreated dental decay and missing and filled teeth (proportion of children with ≥1 decayed teeth, ≥1 missing teeth, and ≥1 filled teeth).

### Descriptive Data

We collected descriptive data at baseline. These included maternal age, education, income, government means-tested health care card status and residential location (sociodemographic characteristics), usual reason for visiting a dentist, maternal toothbrushing behavior, self-rated oral health, and self-rated general health (health status and dental behavior characteristics).

Based on a median split, maternal age was dichotomized into 14 to 24 years vs 25 or more years (median age, 24 years), while education was split into high school or less vs trade/technical or university. Income was characterized by job vs Centrelink (welfare) and owning of a government means-tested health care card (yes vs no). In Australia, welfare payments are made to the unemployed through Centrelink. Metropolitan (Adelaide and outer suburbs) vs nonmetropolitan (regional areas) were the descriptors used to characterize residential location.

“What is your usual reason for seeing a dentist?” and “Did you brush your teeth yesterday?” were the questions used to characterize dental behavior, with response options including problem vs checkup for the former and yes vs no for the latter. The question “How do you think your general/dental health is?” was used to characterize self-rated oral health and general health status. Responses were dichotomized to excellent, very good, or good vs fair or poor.

### Statistical Analysis

Based on an intervention focusing on early childhood caries among Australian Northern Territory–based Indigenous children,^26^ a sample size of 362 (181 in each trial arm) was estimated to be necessary to detect a 20% difference (a clinically meaningful effect size) in early childhood caries prevalence between the 2 groups, at a 2-sided .05 significance criterion and 80% power. Assuming an attrition rate of 20% after 24 months, 450 participants at baseline would be necessary. We recruited 448 at baseline.

Intent-to-treat principles underpinned the data analyses approach. For both the II and DI groups, the number and percentage of participant characteristics at baseline were calculated. General linear regression models with Poisson distribution were used to compare the usefulness of the intervention at child age 3 years (characterized by the mean dt) between the II and DI groups. This was after adjusting for maternal sociodemographic, dental behavior, and health status characteristics at baseline. The mean number of teeth per child in which prevention of dental caries occurred owing to the intervention is characterized by the usefulness estimate. The estimate’s precision is demonstrated by the 95% CIs, with the difference between groups considered to be statistically significant if the 95% CIs do not include zero. Owing to differences reported in the literature of Aboriginal child experience of dental caries by residential location,^27^ we decided a priori to also investigate usefulness according to metropolitan vs nonmetropolitan areas.

### Hypotheses Testing

**Figure 2.**
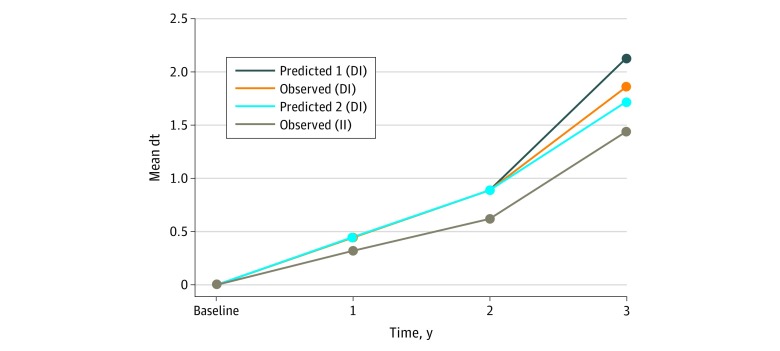
Predicted and Observed Trajectory of the Mean Number of Untreated Caries Between Ages 2 and 3 Years DI indicates delayed intervention; II, immediate intervention; and mean dt, mean number of teeth with untreated decay.

We predicted the mean untreated decay (dt) in the DI group under the following 2 assumptions and scenarios: (1) if the caregiver or child in the DI group did not receive the intervention at all and (2) if the intervention’s effectiveness would be same in both the II and DI groups based on the estimated mean untreated decay (dt) of both groups at 2 and 3 years’ follow-up. We then drew an observed trajectory (Figure 2). We used *t* tests to test the 3 hypotheses. To test for hypothesis 1, the difference in outcome (mean dt) at age 3 years between the II and DI groups was calculated. To test for hypothesis 2, the predicted mean dt (if no intervention was received) within the DI group was compared with the actual mean dt estimates in the II group at age 3 years. To test for hypothesis 3, the predicted mean dt (if received or had the same intervention effect) within the DI group was compared with the actual mean dt estimates in the II group at age 3 years.

Formulas were used for the predicted number of teeth with untreated decay (dt). For predicted 1 (p1), if a caregiver or child in the DI group would not receive the intervention, then the number of dt would be as follows: *Y_i_*_(dt3_p1_DI)_ = *Y_i_*_(dt3_DI)_ + β_NI_ *X* (*i* = *n* = 1,2,3…218, *x* = 1), β_NI_ = 0.89 − 0.62 = 0.27. For predicted 2 (p2), if the intervention effectiveness would be the same in both the II and DI groups, then the number of dt would be as follows: *Y_i_*_(dt3_p2_DI)_ = *Y_i_*_(dt3_DI)_ − β_EI_ *X* (if *Y_i_*_(dt3_DI)_ = 0, then *Y_i_*_(dt3_p2)_ = 0), (*i* = *n* = 1,2,3…218, *x* = 1), β_EI_ = (1.86 − 0.89) − (1.44 − 0.62). Slope was β_NI _and β_EI_. For intercept, *Yi*_(dt3_DI)_ was the observed or estimated mean number of dt in the DI group at 3 years.

To test for hypothesis 1, the difference in outcome (mean dt) at age 3 years between the II and DI groups was calculated after adjusting for other confounders. To test for hypothesis 2, the predicted trajectory of the mean dt at age 3 years within the DI group was compared with the actual dt estimates after receipt of the intervention between ages 2 and 3 years. To test for hypothesis 3, the difference in the mean dt from ages 2 to 3 years was calculated using a paired *t* test after adjusting for confounders.

The proportion of cases able to be prevented if a group takes part in an intervention compared with a group that does not take part is termed the *preventive fraction*. The preventive fraction is obtained by dividing the absolute value of the usefulness estimate by the mean of the dependent variable under consideration in the control group (eg, the number of carious lesions able to be prevented if a group of children is exposed to an intervention compared with a group not exposed). A fully conditional specification method with logistic regression for binary variables and linear regression for continuous variables was used to impute missing data assuming data were missing at random. Missing data from both baseline and outcome variables were imputed, excluding deceased infant outcomes. The II and DI groups were imputed separately. Fifty data sets were imputed using 50 iterations first. Then, the MIANALYZE procedure combined results of the analyses from each imputed data set to generate univariate valid statistical inference.^28^ The primary results are from imputed analyses.

The missing not at random (MNAR) adjustment statement was used in sensitivity analyses to impute our primary outcome variable (untreated dental decay) under the missing at random (MAR) assumption. This included maximum and minimum value imputations, as well as different percentages. The randomization and concealment of allocation processes have been described elsewhere.^21^ To impute and analyze data, we used a statistical software program (SAS, version 9.4; SAS Institute Inc).

## Results

We recruited 448 mothers pregnant with an Aboriginal child between February 1, 2011, and May 30, 2012; of these, 223 were randomly allocated to the II group, and 225 were randomly allocated to the DI group (Figure 1). At baseline, the II and DI groups were well matched, aside from a 10% difference in the frequency of excellent, very good, or good self-rated oral health (higher among the DI group) (Table 1).

**Table 1.** Baseline Maternal Sociodemographic and Dental Behavioral Characteristics

Variable	Baseline No./Total No. (%)^a^		
	Total	II^b^	DI^c^
Total	448	223	225
Maternal age, y			
14-24	283/448 (53.1)	130/223 (58.3)	108/225 (48.0)
≥25	210/448 (46.9)	93/223 (41.7)	117/225 (52.0)
Level of education			
High school or less	322/445 (72.4)	162/221 (73.3)	160/224 (71.4)
Trade/technical or university	123/445 (27.6)	59/221 (26.7)	64/224 (28.6)
Income			
Job	62/443 (14.0)	32/221 (14.5)	30/222 (13.5)
Centrelink^d^	381/443 (86.0)	189/221 (85.5)	192/222 (86.5)
Health care card status^e^			
Yes	358/435 (82.3)	175/214 (81.8)	183/221 (82.8)
No	77/435 (17.7)	39/214 (18.2)	38/221 (17.2)
Residential location			
Metropolitan	171/442 (38.7)	79/220 (35.9)	92/222 (41.4)
Nonmetropolitan	271/442 (61.3)	141/220 (64.1)	130/222 (58.6)
Usual reason for visiting a dentist			
Problem	275/430 (64.0)	141/217 (65.0)	134/213 (62.9)
Checkup	155/430 (36.0)	76/217 (35.0)	79/213 (37.1)
Toothbrushing yesterday			
Yes	321/428 (75.0)	158/213 (74.2)	163/215 (75.8)
No	107/428 (25.0)	55/213 (25.8)	52/215 (24.2)
Self-rated oral health			
Excellent, very good, or good	203/448 (45.3)	90/223 (40.4)	113/225 (50.2)
Fair or poor	245/448 (54.7)	133/223 (59.6)	112/225 (49.8)
Self-rated general health			
Excellent, very good, or good	402/447 (89.9)	197/222 (88.7)	205/225 (91.1)
Fair or poor	45/447 (10.1)	25/222 (11.3)	20/225 (8.9)

Abbreviations: DI, delayed intervention; II, immediate intervention.

^a^ Percentages may not sum to heading totals due to rounding.

^b^ Received intervention at baseline.

^c^ Received intervention after 2-year follow-up.

^d^ Centrelink is the Australian agency that provides welfare payments to those who are unemployed.

^e^ Government means-tested health care card holder.

The first follow-up visit was conducted at child mean age 2 years, with clinical dental data from 324 children (159 II and 165 DI) available. The second follow-up visit was conducted at child mean age 3 years, with clinical dental data from 324 children (157 II and 167 DI) available (52.3% male). Mothers of children lost to follow-up by 3 years compared with those remaining in the study shared largely the same distribution of baseline characteristics. The exceptions were maternal age for children lost to follow-up and self-rated oral health for children at the 3-year follow-up (eTable 1 in the Supplement).

The prevalence of both untreated decay (% dt >0) and dental caries experience (% decayed, missing, or filled teeth [dmft] >0) was more than 8% higher among the DI group than the II group at age 3 years (Table 2). This increased to 9% after adjusting for maternal characteristics at baseline.

**Table 2.** Experience of Dental Disease at Child Age 3 Years by Intervention Group and Residential Location

Variable	Value (95% CI)					
	II	DI	Unadjusted		Adjusted^a^	
			Difference	Prevented Fraction, %	Difference	Prevented Fraction, %
Severity of dental disease						
Mean dt	1.44 (1.38 to 1.50)	1.86 (1.80 to 2.03)	−0.41 (−0.52 to −0.10)	22.0 (5.4 to 28.0)	−0.48 (−0.60 to −0.16)	25.8 (8.6 to 32.3)
Mean dmft	1.48 (1.42 to 1.53)	1.87 (1.79 to 2.03)	−0.39 (−0.43 to −0.15)	20.9 (8.0 to 23.0)	−0.46 (−0.58 to −0.15)	24.6 (8.1 to 31.0)
Mean dt by residential location						
Metropolitan	1.34 (1.24 to 1.43)	1.11 (1.05 to 1.18)	0.19 (−0.28 to 0.65)	16.7 (−58.6 to 25.2)	0.20 (−0.29 to 0.68)	17.7 (−61.3 to 26.2)
Nonmetropolitan	1.50 (1.43 to 1.57)	2.56 (2.46 to 2.66)	−1.00 (−1.73 to −0.27)	39.1 (10.6 to 67.6)	−0.88 (−0.88 to −0.32)	34.5 (12.5 to 34.5)
Mean dmft by residential location						
Metropolitan	1.34 (1.25 to 1.43)	1.12 (1.05 to 1.19)	0.18 (−0.27 to 0.63)	15.9 (−56.3 to 24.1)	0.19 (−0.29 to 0.66)	16.6 (−58.9 to 25.9)
Nonmetropolitan	1.56 (1.48 to 1.63)	2.57 (2.47 to 2.67)	−0.88 (−1.76 to −0.01)	35.5 (0.4 to 68.5)	−1.07 (−1.93 to −0.16)	41.6 (6.2 to 75.1)
Prevalence of dental disease (proportion of risk)						
% dt >0	29.7 (28.8 to 30.5)	37.7 (36.8 to 38.6)	−8.1 (−9.3 to −6.8)	21.3 (18.0 to 24.7)	−8.7 (−9.9 to −7.4)	22.8 (19.7 to 26.2)
% dmft >0	30.1 (29.2 to 31.0)	38.2 (37.3 to 39.0)	−8.1 (−9.3 to −6.8)	21.1 (17.8 to 24.4)	−8.8 (−10.0 to −7.6)	22.8 (19.8 to 26.2)

Abbreviations: DI, delayed intervention; dmft, decayed, missing, or filled teeth; dt, teeth with untreated decay; II, immediate intervention.

^a^ Adjusted for baseline maternal sociodemographic (age, level of education, income, health care card status, and residential location), dental behavior (usual reason for visiting a dentist and toothbrushing yesterday), and health status (self-rated oral and general health) characteristics.

### Hypothesis 1

At child age 3 years, the mean dt was 1.44 (95% CI, 1.38-1.50) for the II group and 1.86 (95% CI, 1.89-2.03) for the DI group (Table 2); the differences were statistically significant. The unadjusted model estimates showed that the II group had −0.41 (95% CI, −0.52 to −0.10) lower levels of noncavitated plus cavitated lesions compared with the DI group. The predicted mean dt at age 3 years for the DI group was 2.15. The prevented fraction in the unadjusted model was 20%. After adjusting for baseline covariates, the usefulness estimate increased slightly to −0.48 for noncavitated plus cavitated lesions, with a prevented fraction of 25.8%. When examining the overall experience of dental disease, the II group had −0.39 lower levels of dmft compared with the DI group. The prevented fraction in the unadjusted model was 20.9%. After adjusting for covariates, the usefulness estimate increased to −0.46 for dmft, with a prevented fraction of 24.6%. Children residing in metropolitan regions had significantly less experience of dental disease than children residing in nonmetropolitan settings at child age 3 years, regardless of intervention group. The mean dt of those in the II group was significantly less than that in the DI group (1.50 compared with 2.56, a mean difference of −1.00 tooth, with a prevented fraction of 39.1%) when considering the nonmetropolitan-dwelling children in isolation. Results were just as stark when considering the overall experience of dmft, with those in the II group having a mean dmft of 1.56 compared with 2.57 in the DI group, a mean difference of −0.88, with a prevented fraction of 35.5%, which increased to 41.6% after adjusting for maternal baseline characteristics. The proportion of children with both untreated dental decay (% dt >0) and experience of dental disease (% dmft >0) was −8.1% lower in the II group compared with the DI group, which increased slightly for both outcomes to 8.7% and 8.8%, respectively, after adjustment. The preventive fraction (for both % dt >0 and % dmft >0) was 21.3% unadjusted and 22.8% adjusted.

At child age 3 years, the prevalence of untreated dental caries and the number needed to treat (NNT) are listed in Table 3. The NNT to prevent 1 child from developing caries (noncavitated plus cavitated lesions) was 12.4 when all children were considered together. This was highest among children living in metropolitan areas (85.5) and lowest among mothers with fair or poor self-rated general health at baseline (5.1).

**Table 3.** Caries Prevalence^a^ and Number of Children Needed to Treat (NTT) at 3 Years’ Follow-up

Variable	% (95% CI)		*P* Value	NNT
	II^b^	DI^c^		
All children	29.7 (28.8-30.5)	37.7 (36.8-38.6)	<.001	12.4
Maternal age, y				
14-24	31.1 (29.6-32.4)	34.2 (32.7-36.2)	<.001	32.3
≥25	27.7 (26.7-29.3)	41.0 (38.8-41.3)	<.001	7.5
Education				
High school or less	34.3 (33.1-35.6)	44.3 (41.7-46.4)	<.001	10.0
Trade/technical or university	17.4 (16.1-19.1)	20.8 (19.8-22.9)	.001	29.4
Income				
Job	20.8 (18.6-23.4)	14.1 (12.1-16.2)	<.001	14.9
Centrelink^d^	31.2 (29.3-31.8)	41.4 (40.1-43.1)	<.001	9.8
Health care card status^e^				
Yes	31.0 (29.5-31.8)	42.1 (40.1-42.5)	<.001	9.0
No	23.8 (22.3-26.4)	16.7 (14.8-18.7)	<.001	14.0
Residential location				
Metropolitan	28.4 (26.4-30.1)	29.5 (27.5-31.5)	.24	85.5
Nonmetropolitan	30.4 (28.8-31.3)	42.5 (40.7-43.4)	<.001	7.6
Usual reason for visiting a dentist				
Problem	35.9 (34.1-36.6)	37.8 (36.9-39.5)	.02	53.5
Checkup	18.2 (16.2-20.2)	37.6 (35.6-39.6)	<.001	5.2
Toothbrushing yesterday				
Yes	27.1 (25.4-29.1)	31.7 (30.1-34.0)	<.001	22.1
No	36.7 (34.6-39.2)	55.5 (53.3-57.8)	<.001	5.3
Self-rated oral health				
Excellent, very good, or good	29.2 (27.0-31.1)	25.3 (24.0-26.6)	.03	50.5
Fair or poor	29.9 (26.0-29.6)	48.3 (45.8-48.5)	<.001	5.4
Self-rated general health				
Excellent, very good, or good	31.0 (28.6-31.4)	37.6 (36.9-39.0)	<.001	15.2
Fair or poor	19.0 (17.2-21.4)	38.7 (34.2-42.3)	<.001	5.1

Abbreviations: DI, delayed intervention; dt, teeth with untreated decay; II, immediate intervention.

^a^ Percent of dt greater than 0.

^b^ Received intervention at baseline.

^c^ Received intervention after 2-year follow-up.

^d^ Centrelink is the Australian agency that provides welfare payments to those who are unemployed.

^e^ Government means-tested health care card holder.

### Hypothesis 2

The estimated untreated dental caries was less in the DI group after receipt of the intervention between ages 2 and 3 years than if no intervention had been received (Figure 2). The predicted mean dt at age 3 years was 2.13, but the actual mean dt was 1.86. The mean difference compared with the II group was −0.70 (95% CI, −1.32 to −0.11; *P* = .02).

The estimated untreated dental caries was more in the DI group after receipt of the intervention between ages 2 and 3 years than if the same intervention was received earlier in infancy (Figure 2). The predicted mean dt at age 3 years was 1.72 (95% CI, 1.65-1.77), with a difference of −0.26 (95% CI, −0.47 to −0.05; *P* = .02).

### Hypothesis 3

Between ages 2 and 3 years, the caries increment for the II group was 0.82 (95% CI, 0.75-0.89). This is compared with 0.97 (95% CI, 0.87-1.17) for the DI group (*P* = .05).

In eTable 2 in the Supplement, we summarize results from the sensitivity analyses, including imputed data for untreated dental caries, unimputed data, and covariate-adjusted differences in the means and rate ratios. Although findings were largely consistent with those from the primary analyses, there were some exceptions (eTable 2 and eTable 3 in the Supplement).

## Discussion

We tested 3 hypotheses in this study. These include that (1) usefulness of a standardized, robustly structured, culturally safe, and carefully administered early childhood caries trial delivered to an Australian Aboriginal population between pregnancy and child age 18 months would be demonstrated at the 3-year follow-up; (2) the trajectory of untreated dental decay among children in the DI group would be lower than predicted after receipt of the intervention at age 2 years; and (3) the intervention would be more effective (ie, the increment in untreated dental caries between the 2-year and 3-year follow-ups) in pregnancy or early infancy than in later infancy or early childhood.

Our first hypothesis proved true. At the 3-year follow-up, children in the II group who received the intervention during pregnancy or early infancy had statistically less clinically detected untreated dental caries than their counterparts in the DI group. In other words, approximately four-fifths of children in the II group had no experience of dental caries at age 3 years compared with only two-thirds of children in the DI group. Our second hypothesis also proved true, with children in the DI group developing further dental caries between ages 2 and 3 years, but at a lower trajectory than that predicted had the intervention not been received. Our third hypothesis also proved true; the caries increment was less between ages 2 to 3 years among children in the II group compared with the DI group.

### Strengths and Limitations

Our study has numerous strengths. These include strict adherence to (and success of) the randomization process, inclusion criteria that were broad in their scope (any woman pregnant with an Aboriginal child), appropriate sample size, extensive follow-up, and masking of those involved in outcome assessments. We may have missed child death recordings, and there were some missing data on outcome items, meaning that internal validity may have been weakened. Furthermore, it was not possible to obtain data from administrative sources on postnatal characteristics that might have influenced the primary outcome (eg, trauma experienced in the home, removal of children, incarceration of family members, and death). However, the randomization process was likely to have distributed factors external to the trial evenly among the II and DI groups, although we appreciate that this does not always hold true. In addition, our outcome data held true after sensitivity analyses, and there were no concerning differences between the baseline characteristics of those lost to follow-up and those who remained in the study. We consider our findings robust, representative, and suggestive of an association between a culturally safe and context-specific oral health intervention and improved and sustained child oral health outcomes for the following 3 main reasons: (1) biological plausibility, (2) consistency among both a range of indicators and time, and (3) constant strength of association.

The main limitation of the study is that the influence of the third intervention delivered at 36 months to the DI group (ie, application of fluoride varnish to the teeth of children and combined anticipatory guidance and MI session with the caregiver) could not be evaluated. The reason is because this was the same time point at which clinical outcomes were assessed.

Therefore, the study results support the hypothesis that an early childhood caries intervention delivered earlier rather than later in infancy confers greater benefits. However, it is worth highlighting that the overall prevalence of untreated dental decay was still much higher than reports among the general child population in South Australia.^29^ Although encouraging, these results should not be interpreted to be effective among all Aboriginal child groups in Australia, who each have important geographical, historical, and contextual differences.

During the study duration, there were no secular trends in Aboriginal child dental health outcomes in the study area or major changes to dental health policy or structuring. However, the recommendation for fluoride varnish to be implemented by nondental staff was mandated in many Australian jurisdictions during our analyses phase. In addition, caregivers of Aboriginal children in South Australia are now eligible for free or low-cost publicly funded dental care through the South Australian Dental Service, including a waiting list waiver and transportation services. This was not available at the time of our study.

## Conclusions

Our findings suggest the following: (1) that at the 3-year follow-up children in the II group who received the intervention during pregnancy or early infancy had statistically less clinically detected untreated dental caries than their counterparts in the DI group, (2) that children in the DI group developed dental caries at a lower trajectory than predicted had the intervention not been received at ages 2 to 3 years, and (3) that the caries increment was less between ages 2 and 3 years among children in the II group compared with the DI group, indicating that the best time to implement the intervention is earlier rather than later infancy. Our findings have value for Aboriginal Community Controlled Health Organisations in Australia with an interest in including dental care and oral health promotion in their scope of health service provision and indeed at an international level because Indigenous children worldwide are recognized as having poorer oral health outcomes than their non-Indigenous peers.

## Notes

Supplement. **eTable 1.** Baseline Sample Characteristics by Follow-up and Loss-to-Follow-up Children at 3-Year Examinations **eTable 2.** Sensitivity Analyses Under Different Scenarios **eTable 3.** Test for Hypotheses
