# Supplementary material for: Follow-up of an Intervention to Reduce Dental Caries in Indigenous Australian Children: A Secondary Analysis of a Randomized Clinical Trial
Source: JAMA Netw Open. 2019 Mar 15;2(3):e190648. doi: 10.1001/jamanetworkopen.2019.0648 (PMC6484654; doi:10.1001/jamanetworkopen.2019.0648)
Supplement: Supplement. — eTable 1. Baseline Sample Characteristics by Follow-up and Loss-to-Follow-up Children at 3-Year Examinations eTable 2. Sensitivity Analyses Under Different Scenarios eTable 3. Test for Hypotheses [file jamanetwopen-2-e190648-s001.pdf]

Supplementary Online Content

Jamieson LM, Smithers LG, Hedges J, et al. Follow-up of an intervention to reduce dental caries in indigenous Australian children: a secondary analysis of a randomized clinical trial. *JAMA Netw Open*. 2019:2(3):e190648. doi:10.1001/jamanetworkopen.2019.0648

eTable 1. Baseline Sample Characteristics by Follow-up and Loss-to-Follow-up Children at 3-Year Examinations

eTable 2. Sensitivity Analyses Under Different Scenarios

eTable 3. Test for Hypotheses

This supplementary material has been provided by the authors to give readers additional information about their work.

© 2019 Jamieson LM et al. *JAMA Network Open*.

eTable 1. Baseline Sample Characteristics by Follow-up and Loss-to-Follow-up Children at 3-Year Examinations

	**3 year follow-up**					**3 year loss to follow-up**			
	**N (%)**			***p-value**		**N (%)**			**p-value**
Variables	Total (n=324)	Immediate Intervention (n=157)	Delayed Intervention (n=167)			Total (n=112)	Immediate Intervention (n=61)	Delayed Intervention (n=51)	
**Maternal age**				0.446					0.003
14-24	173 (53.2)	87 (55.4)	86 (51.2)			59 (52.7)	40 (65.6)	19 (37.3)	
25+	152 (46.8)	70 (44.6)	82 (48.8)			53 (47.3)	21 (34.4)	32 (62.8)	
**Education**				0.840					0.566
High school or less	232 (72.1)	111 (71.6)	122 (72.6)			82 (73.2)	46 (75.4)	36 (70.6)	
Trade or University	90 (28.0)	44 (28.4)	46 (27.4)			30 (26.8)	15 (24.6)	15 (29.4)	
**Income**				0.296					0.173
Job	55 (17.1)	30 (19.4)	25 (15.0)			5 (4.5)	1 (1.6)	4 (8.0)	
Centrelink	267 (82.9)	125 (80.7)	142 (85.0)			106 (95.5)	60 (98.4)	46 (92.0)	
**HCC status**				0.710					0.864
Yes	252 (79.3)	120 (78.4)	133 (80.1)			97 (91.5)	51 (8.9)	46 (92.0)	
No	66 (20.8)	33 (21.6)	33 (19.9)			9 (8.5)	5 (8.9)	4 (8.0)	
**Residential location**				0.138					0.869
Metropolitan	116 (36.1)	50 (32.1)	66 (40.0)			51 (46.0)	28 (46.7)	23 (45.1)	
Non- metropolitan	205 (63.9)	106 (68.0)	99 (60.0)			60 (54.1)	32 (53.3)	28 (54.9)	
**Usual reason visit dentist**				0.5490					0.664
Problem	194 (62.4)	98 (64.1)	96 (60.8)			74 (67.9)	39 (66.1)	35 (70.0)	
Check-up	117 (37.6)	55 (36.0)	62 (39.2)			35 (32.1)	20 (33.9)	15 (30.0)	
**Brush yesterday**				0.759					0.982
Yes	240 (77.4)	115 (76.7)	125 (78.1)			74 (67.9)	40 (67.8)	34 (68.0)	
No	70 (22.6)	35 (23.3)	35 (21.9)			35 (32.1)	19 (32.2)	16 (32.0)	
**Self-rated oral health**				0.027					0.428
Excellent, very good or good	151 (46.5)	63 (40.1)	88 (52.4)			46 (41.1)	23 (37.7)	23 (45.1)	
Fair or poor	174 (53.5)	94 (59.9)	80 (47.6)			66 (58.9)	38 (62.3)	28 (54.9)	
**Self-rated general health**				0.310					0.919
Excellent, very good or good	298 (92.0)	141 (90.4)	157 (93.5)			94 (83.9)	51 (83.6)	43 (84.3)	
Fair or poor	26 (8.0)	15 (9.6)	11(6.6)			18 (16.1)	10 (16.4)	8 (15.7)	

* Chi-square test, and cell number <5 Fisher's Exact test

© 2019 Jamieson LM et al. *JAMA Network Open*.

eTable 2. Sensitivity Analyses Under Different Scenarios

Scenarios	Intervention effect (Mean difference)	95% CI		Pr > |t|
1	-0.31	-0.49	-0.13	0.001
2	-0.30	-0.47	-0.13	0.000
3	-0.29	-0.46	-0.12	0.001
4	-0.27	-0.44	-0.11	0.001
5	-0.26	-0.43	-0.10	0.002
6	-0.22	-0.38	-0.05	0.011
7	-0.25	-0.42	-0.07	0.005
8	-0.23	-0.40	-0.05	0.013
9	-0.21	-0.38	-0.04	0.017
10	-0.23	-0.41	-0.04	0.017
11	-0.22	-0.40	-0.04	0.016
12	-1.37	-1.53	-1.22	0.000
13	-1.26	-1.42	-1.09	0.000
14	1.87	1.36	2.39	0.000
15	1.06	0.93	1.19	0.000
16	-0.21	-0.40	-0.02	0.029
17	0.04	-0.04	0.12	0.365
18	0.02	-0.07	0.12	0.632

Notes: Missing data ranged from 1.8% to 4.5% ('Brushing yesterday') for baseline variables, outcome variable (dt) was 25.9%. Therefore, the sensitivity analyses were based on dt1 to do MI (Max. dt=17 in this study, so we tested both Max. dt=17 and Max.dt =20).

a. Scenario 1-11 we multiplied the imputed values of dt1 by 0%, 10%, 20%, 30%, 40%, 50%, 60%, 70%, 80%, 90% and 100% at each step of the iterative process under the MNAR ADJUST statement (scale: 0, 0.1, 0.2, 0.3, 0.4, 0.5, 0.6, 0.7, 0.8, 0.9 and 1, respectively).
b. Scenario 12-13 we imputed values of dt: dt= 0 in the immediate intervention group and dt=20, and dt=17 in delayed intervention group, respectively.
c. Scenario 14-15 we imputed values of dt: dt= 20, and dt=17 in the immediate intervention group, and dt=0 in delayed intervention group, respectively.
d. Scenario 16 we imputed values of dt: dt= 0 in both immediate and delayed intervention groups.
e. Scenario 17-18 we imputed values of dt: dt=20 and dt=17 in both immediate and delayed intervention groups, respectively.

© 2019 Jamieson LM et al. *JAMA Network Open*.

eTable 3. Test for Hypotheses

	Immediate	Delayed			Difference	P-Value
**Hypotheses**	Observed	Observed	Predicted 1	Predicted 2		
	Mean dt (95% CI)				ΔMean (95% CI)	
Hypothesis 1:	1.44 (1.38,1.50)	1.86 (1.80, 2.03)			-0.41 (-0.52, -0.10)	0.046
Hypothesis 2:	1.44 (1.38,1.50)		2.13 (2.07, 2.19)		-0.70 (-1.32, -0.11)	0.024
Hypothesis 3:	1.44 (1.38,1.50)			1.72 (1.65, 1.77)	-0.26 (-0.47, -0.05)	0.021

Notes: Formulas for predicted number of untreated decay (dt):

Predicted 1: If caregiver-child of the DI group would not receive the intervention, then the number of dt would be: $Y_{i(dt3\_p1\_DI)}=Y_{i(dt3\_DI)}+\beta_{NI}X$ (i=n=1,2,3…218, x=1), $\beta_{NI}=0.89-0.62=0.27$

Predicted 2: If the intervention effective would be same in the both II and DI intervention groups, then the number of dt would be: $Y_{i(dt3\_p2\_DI)}=Y_{i(dt3\_DI)}-\beta_{EI}X$ (If $Y_{i(dt3\_DI}=0$, then $Y_{i(dt3\_p2)}=0$), (i=n=1,2,3…218, x=1), $\beta_{EI}=(1.86-0.89)-(1.44-0.62)$

Slope: β_{NI} and β_{EI}

Intercept: $Y_{i(dt3\_DI)}$ was observed/estimated mean number of dt in delated group at 3 years

© 2019 Jamieson LM et al. *JAMA Network Open*.
